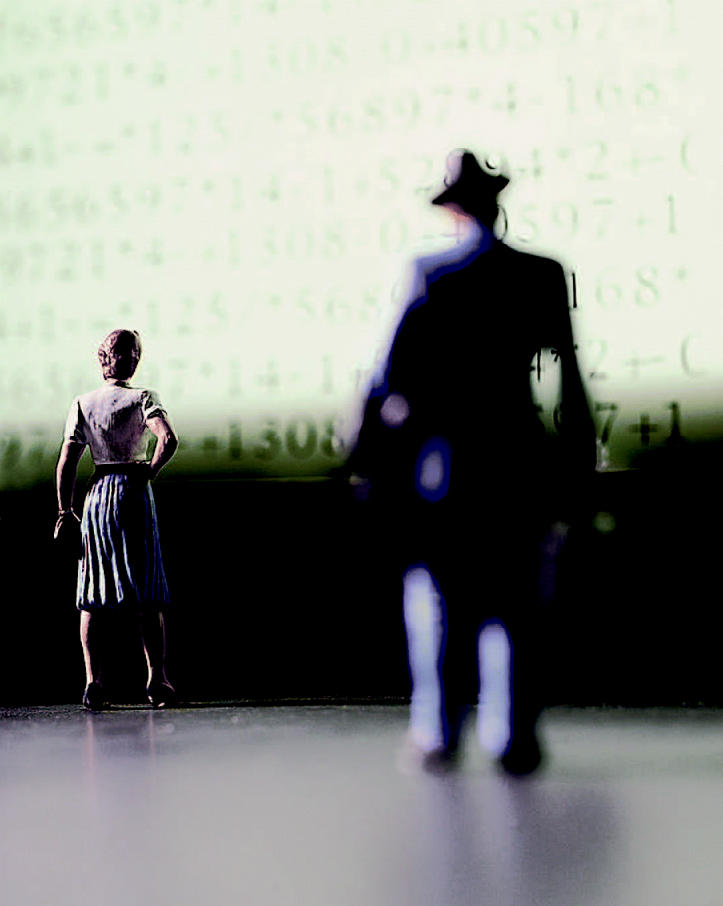# The Regulation Equation: Factoring In the Price of Health

**DOI:** 10.1289/ehp.114-a296

**Published:** 2006-05

**Authors:** Bob Weinhold

Controversy erupted early in 2003 after the U.S. Office of Management and Budget (OMB) proposed that the lives of older people were worth less in dollar terms than those of younger people. The idea was included in a plan published in the 3 February 2003 *Federal Register* by the OMB’s Office of Information and Regulatory Affairs (OIRA) that was designed to improve how the federal government determines the benefits and costs of proposed regulations, including environmental regulations. A revised version issued 17 September 2003, called Circular A-4, stipulates that specific age-adjustment factors should not be used. But it still includes a number of calculation processes that many perceive discount the value of health as people age.

To help address the controversy that still simmers over how, or whether, to assign a specific value to effects such as degraded human health, OIRA and several federal agencies asked a committee of the National Academies’ Institute of Medicine (IOM) to weigh in with guidance on one type of cost–benefit analysis, called cost-effectiveness analysis (CEA), which can include calculations of the dollar value of human life and which was included in Circular A-4. After an effort spanning about two years, the committee issued its report, *Valuing Health for Regulatory Cost-Effectiveness Analysis*, on 11 January 2006.

The committee concluded that the techniques advocated by the OMB, including CEA, have their place, but also have important deficiencies, which could be addressed to some extent by following the committee’s main recommendations. In addition, the committee—whose 16 members represent several U.S. and Canadian universities, health care systems, and state and federal agencies—cautions that CEA likely will remain an imperfect tool that should be balanced with other objective and subjective considerations of a regulation’s impact.

Uncertainties about the future use of CEA, as well as the OMB’s overall regulatory review approach, continue to stir sharp divisions among critics and supporters. All sides are closely watching the OMB to see how it proceeds.

## Calculating All Effects

OIRA oversees the implementation of many governmentwide policies, including the adoption of new regulations. For regulations, its emphasis is on impact analysis, particularly of economic impacts, as well as interagency coordination of regulations and consideration of alternative rules and regulatory approaches.

Under former administrator John Graham, the OIRA emphasized the importance of cost–benefit analysis when reviewing proposed federal agency regulations that had to funnel through his office. Cost–benefit analysis looks at dollars gained and spent in both the public and private sectors as the result of a regulation.

However, some regulatory impacts—such as effects on human health—are difficult, if not impossible, to express in dollars. As a result, OIRA also began to emphasize CEA, which attempts to account for effects like these by assigning a number, tied to some kind of synthetic index, to the benefit side of the equation. This number reflects impacts such as tons of pollutants reduced or years of life gained. CEA has been evolving for several decades in the medical field, but is in its relative infancy when applied to other areas.

OIRA laid out its version of CEA requirements in Circular A-4, and said its analytical process had to be used for any proposed regulation estimated to have an annual effect on the economy of $100 million or more. The IOM committee found that only 18 regulations meeting that standard were finalized in the period from January 2000 to June 2004, out of thousands of federal rules proposed every year. Among these were the EPA’s efforts to address diesel engine emissions and arsenic in drinking water, an FDA regulation on juice processing contaminants, and a Food Safety and Inspection Service regulation on *Listeria* contamination in meat and poultry. The committee says a number of upcoming regulations likely will need to complete a CEA.

## New Ways to Crunch the Numbers

The committee made a dozen primary recommendations to improve the use of CEA. Many of these address exactly how a CEA should be conducted. For instance, the committee recommends the use of a measure called a quality-adjusted life year, or QALY, to create the most viable measure of human health impacts. Calculations of QALYs address both length of life and degradation of health to create a score. However, the committee says even this widely used tool has limited data supporting it, and much more information must be developed to improve it.

One way to do this is to acquire better baseline information by adding appropriate questions to and coordinating better among existing national surveys, such as the National Health Interview Survey and the Medical Expenditure Panel Survey. This would provide a better perspective on how the general public judges various health outcomes. For example, how would someone score the effects of short-term arthritis versus long-term arthritis that waxes and wanes but never resolves?

Some research is already under way on the half dozen most commonly used questionnaires designed to gauge individual judgments on health impacts. A team led by IOM committee member Dennis Fryback, a professor of population health sciences at the University of Wisconsin–Madison, is trying to develop a “Rosetta stone” that will aid comparison between the sometimes-disparate results from different questionnaires, increasing their statistical power. Based on three studies of about 3,900 U.S. residents, Fryback hopes to begin presenting results late in 2006, with journal publication through 2007 and early 2008.

The committee also recommended that improved regulatory analysis should include clearer and more prominent explanations of the many uncertainties inherent in CEAs; should better address differences in impacts on various geographic areas and groups, such as infants, the elderly, and those of different races and economic classes; should be standardized so that all federal agencies use a common approach; and should be more transparent and open to public involvement and review.

## A League of Their Own

Even with these recommendations, a CEA unavoidably has to put a price on the health impacts and regulatory costs involved in saving a QALY—that is, how much are we, as individuals and as a society, willing to pay per unit of gained healthy life? Controversy over that concept may increase in the future, since one OMB goal has been to use CEA and cost–benefit analysis to develop tools called “league tables.”

Similar to sports league standings, league tables could provide a simple way to compare regulations, even if they cover diverse topics. A regulation to cut *Escherichia coli* in food might be reduced to a score of 27, while a regulation to slash auto accident fatalities might have a score of 39, and a regulation to throttle sulfur dioxide pollution might have a score of 62. (These numbers are purely hypothetical, for the sake of example, since the OMB has not yet developed accepted scales for scoring.)

This strategy fits within OMB’s broader objective to adopt “regulatory budgeting,” which includes the idea that when all public and private parties meet a preset dollar figure assigned to regulatory expenditures each year, no more regulations can be passed. These approaches are desirable, says Angela Logomasini, director of risk and environmental policy at the Competitive Enterprise Institute, a free enterprise advocacy group, since government needs a tool to decide where best to spend limited resources.

However, the IOM report specifically warns against computing league tables across regulations or areas of regulation, noting that what is considered a benefit and what should be counted as a cost differs from analysis to analysis. “It is analogous to looking at prices of cars where one does not know whether they are comparably equipped, have similar efficiency, and so on,” says Fryback. “We can say that the price per car varies, and that one looks more expensive than another, but without the details these comparisons may be misleading.”

Furthermore, such important determinations can’t rely solely on a tool such as a CEA, says Amy Sinden, an associate professor at Temple University’s Beasley School of Law and a member scholar of another advocacy group, the Center for Progressive Reform. “There’s just not enough data,” she says. “Important aspects of ecological and human health impacts that can’t be quantified get left out. A CEA produces numbers that create an aura of scientific objectivity but that may be misleading. The numbers tell only part of the story.” The worry, she adds, is that when agencies use methods like these, often all the public sees are the numbers, not the nuances.

## The Unknown Factor

The future of OMB’s approach is uncertain. Graham left OIRA and assumed the role of dean of the Frederick S. Pardee RAND Graduate School on 1 March 2006. His permanent successor had not been named as of mid-April 2006. Robert Shull, director of regulatory policy at the nonprofit OMB Watch, suspects the general direction of OMB and OIRA won’t change much, regardless of who is administrator, given that the general direction has already been set by the Bush administration.

IOM committee chairman Robert Lawrence, a professor of preventive medicine at the Johns Hopkins Bloomberg School of Public Health, says that, although initial response by OMB and numerous federal agencies to the report has been good, prospects for specific revisions to current efforts and policies are unclear. Much will be determined by the new OIRA administrator, he says, and many of the affected agencies told him it would be difficult in this budget climate to get additional money to proceed with the committee’s recommendations.

Whatever the outcome, even supporters of the OMB approach realize such measures are less than perfect. “All of these things are highly subjective,” Logomasini says. “Such regulatory reforms are often not as effective as we would like them to be. Ultimately, deciding whether or how to regulate is a policy decision.”

## Figures and Tables

**Figure:**
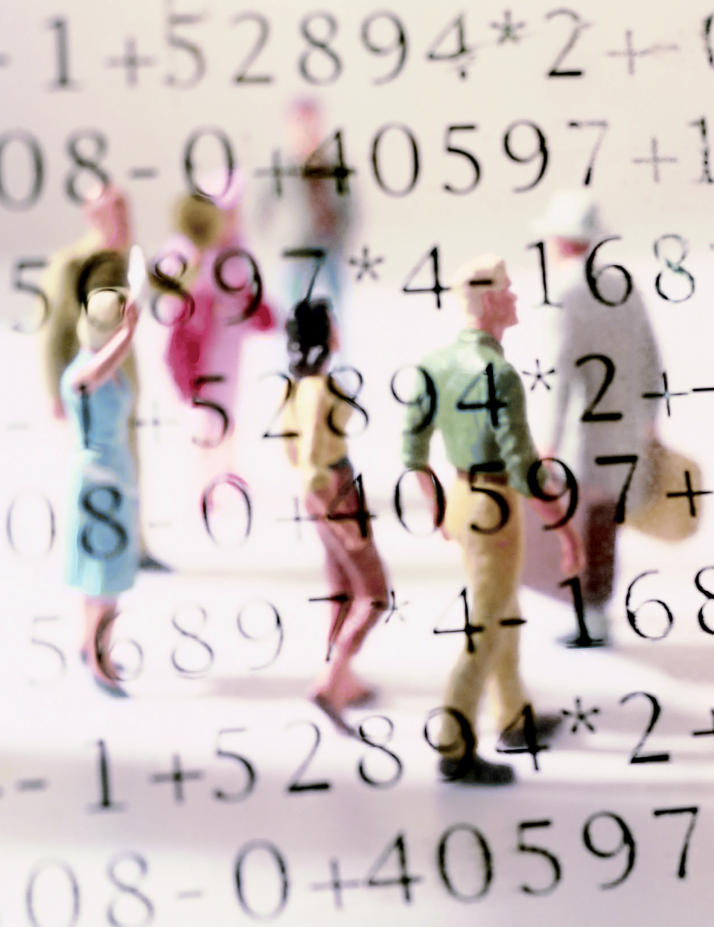


**Figure:**